# Empowering Educators to Build AI Chatbots in Health Professions Education: Tutorial on a No-Code Design Workflow

**DOI:** 10.2196/103399

**Published:** 2026-08-14

**Authors:** Charlene Xing Le Goh, Eugene Wang, Juanita S M Kong, Judy C G Sng

**Affiliations:** 1 Yong Loo Lin School of Medicine, National University of Singapore, Singapore, Singapore; 2 Department of Pharmacology, Yong Loo Lin School of Medicine, National University of Singapore, 16 Medical Drive MD3 Level 4Singapore117600, Singapore, 65 65163676

**Keywords:** artificial intelligence, AI, chatbot, no code, health professions education, virtual patient, prompt engineering, medical education, tutorial, generative AI

## Abstract

AI is entering clinical practice faster than health professions curricula can teach it, leaving many educators eager to use AI-based teaching tools but unsure of how to build them. Generative AI chatbots—configured as simulated patients, clinical coaches, or formative assessment partners—offer scalable, interactive practice without any programming, yet most educators lack a structured method for designing and deploying them well. This tutorial provides that method: a practical, platform-agnostic workflow for building no-code AI chatbots using widely available large language model platforms. The workflow is organized in five sequential sections that follow the arc of a design project: (1) defining the educational purpose, learner group, and persona; (2) configuring behavior through the system prompt, graduated information disclosure, and structured feedback; (3) refining the learner experience through communication-style calibration, voice interaction, and curated knowledge documents; (4) adding realism and testing, including AI avatar generation and rigorous pilot-testing; and (5) embedding the tool within the curriculum and governing its use ethically. Each section pairs concrete, copy-ready design steps with the reasoning behind them, drawing on the technological pedagogical content knowledge framework and on established learning mechanisms—deliberate practice, self-regulated learning, formative feedback, and simulation-based learning—so that design choices are pedagogically grounded rather than merely technical. Throughout, 2 locally developed initiatives, the Virtual Integrated Patient and the Depression Avatars project, serve as illustrative implementation examples that motivated specific design decisions. These are presented as feasibility and acceptability experiences, not as evidence of educational effectiveness. The tutorial also addresses when a chatbot is not the right tool, the principal risks (hallucination, automation bias, data privacy exposure, and bias in generated personas), and a practical governance checklist for safe deployment. Although the examples are clinical, the workflow is discipline-agnostic and transferable across higher education. No-code AI chatbots are a feasible, accessible way for educators to build interactive learning tools; rigorous, multi-institutional evaluation using validated instruments remains the essential next step.

## Introduction

The integration of AI into health care has outpaced the formal training offered in nursing and medical education programs [1]. At the same time, surveys of health professions students reveal strong enthusiasm for AI-assisted learning, with most believing that AI tools will contribute to improved patient outcomes, new research breakthroughs, and greater efficiency in clinical practice (TJ Wong, unpublished data, January 2025). This paradox—students eager for AI-enhanced education within systems slow to adopt it—creates both an imperative and an opportunity for individual educators to act.

One accessible entry point is the AI chatbot. Unlike purpose-built clinical simulation platforms that require institutional infrastructure and technical teams, modern large language model (LLM)–powered chatbots can be configured by educators themselves using no-code interfaces. These tools allow for the construction of interactive learning experiences (simulated patients, clinical coaches, and formative assessment partners) without a single line of programming. The educator’s role shifts from passive consumer to active designer, a transition that aligns with emerging frameworks for the evolving role of teachers in an AI-enabled environment [2].

This tutorial is scoped specifically to generative AI (GenAI) platforms—tools powered by LLMs with a configurable instruction or system prompt field (see Table 1 for representative examples). Rule-based or menu-driven chatbot builders, which operate on predefined decision trees, require different design approaches and are outside the scope of this tutorial.

**Table 1.** Representative no-code tools for educators (as of 2025). This list is representative, not exhaustive. Platform features, pricing tiers, and regional availability change frequently. Educators are advised to verify current terms of service, data sharing policies, and institutional licensing agreements before deployment.

Category and tool	Free tier	Key feature	Educator notes
LLM^a^ chatbot platforms			
ChatGPT (OpenAI)	Yes (limited)	Custom GPT^b^ builder; voice mode; knowledge upload	Custom GPT builder requires a paid plan; most widely used in education worldwide
Microsoft Copilot	Yes (institutional)	Integrated with Microsoft 365; voice input; enterprise privacy	Free via many institutional Microsoft licenses; strong data governance options
Google Gemini	Yes (limited)	Multimodal input; Google Workspace integration	Custom persona Gems available on paid plans
Claude (Anthropic)	Yes (limited)	Large context window; strong instruction following	Projects feature enables persona definition and document grounding
DeepSeek	Yes	Strong reasoning; accessible where other platforms are restricted	Open-source model; data privacy policies vary by deployment context
Avatar and video tools			
D-ID	Trial only	Text-to-avatar video; emotion and movement controls	Used in the NUS^c^ Depression Avatars pilot; photorealistic output; credit-based pricing
HeyGen	Trial only	Photo and video cloning; voice cloning; multilingual translation	Strong multilingual support; widely adopted for educational video production
Synthesia	Trial only	Studio-quality avatars; slide integration; team sharing	Well suited for institutional-scale educational video production
Voice-specific tools			
ElevenLabs	Yes (limited)	High-fidelity voice synthesis and voice cloning	Can generate culturally appropriate patient voices for avatar scripts
NotebookLM (Google)	Yes	Audio overview mode from uploaded documents	Useful for converting course materials into spoken summaries for learners

a LLM: large language model.

b GPT: generative pretrained transformer.

c NUS: National University of Singapore.

While the examples throughout are drawn from health professions education—where patient simulation, clinical communication, and diagnostic reasoning present distinctive design challenges—the underlying workflow is discipline agnostic. Educators in law, engineering, social work, and the humanities will find the core principles equally applicable, substituting clinical scenarios with discipline-appropriate cases.

The workflow is presented in 5 sequential sections summarized in Table 2. Each section addresses a distinct phase of the design and deployment process, from initial planning to ethical stewardship of the deployed tool. A complete step-by-step build guide covering the text-based, voice-enabled, and avatar workflows described here, including ready-to-copy prompts and platform-specific instructions, is provided in Multimedia Appendix 1.

**Table 2.** The no-code chatbot design workflow: 5 sections at a glance. Each row states a general design principle; items marked as “Example” are illustrations drawn from health professions education. The principles are discipline agnostic, and the specific attributes named (eg, a patient’s presenting concern) apply only to the persona type illustrated—educators in other fields should substitute the attributes relevant to their own context.

Section number	Section name	Focus	Design decisions covered
1	Before you build	Defining the purpose, learner, and persona	Define the specific learning outcomes, target learner group, and stage of training. Apply the TPACK^a^ framework to align technology, pedagogy, and content before opening any platform. Specify the chatbot’s identity in detail. Richly specified personas produce consistent, believable interactions; vague ones produce generic outputs. The attributes worth specifying depend on the persona type. Example (simulated patient): demographics, presenting concern, personality, cultural context, and communication style; a coaching, assessor, or nonclinical persona would specify a different set of attributes.
2	Configuring the chatbot	System prompts, disclosure, and feedback	Craft the system prompt (variously labeled instructions, system prompt, or custom instructions across platforms) to specify the role, interaction rules, prohibited behaviors, and tone. This is the educator’s most consequential technical decision. Decide how much the chatbot should volunteer vs withhold. In cases in which the persona simulates an information source, configure graduated disclosure so that details emerge incrementally and only in response to direct questions rather than all at once. Example: a simulated patient reveals symptoms only when asked, mirroring an authentic clinical encounter; a simulated client or stakeholder behaves analogously. Build in structured feedback from the outset, anchored to learning objectives. Use a consistent framework (eg, “What went well / What to improve / Try again”) and include an end-of-session reflective summary.
3	Refining the experience	Learner calibration, voice, and knowledge	Calibrate vocabulary, scaffolding level, response length, and cultural register to match the target learner. Voice-enabled chatbots require additional brevity and single-question turns. Use voice mode deliberately: it increases ecological validity for communication skill training but demands specific design constraints and institutional access verification. Upload concise, structured knowledge documents (guidelines, rubrics, and case files) to ground responses in curriculum-specific content rather than generic model outputs.
4	Adding realism and testing	Avatars, multimodal elements, and pilot-testing	Incorporate AI-generated avatar videos to add visual and emotional authenticity. Generate personas from descriptive text prompts; expect and budget for multiple iterations to achieve satisfactory facial expression and demographic representation. Pilot-test rigorously before deployment: subject matter expert review for content accuracy, then learner testing under realistic conditions. Refine failure modes (overdisclosure, excessive length, and persona drift) by adjusting the system prompt.
5	Embedding and sustaining the tool	Curriculum integration and ethics	Situate the chatbot within a planned instructional sequence—after foundational teaching and before or alongside supervised real-world encounters—and map explicitly to program-level outcomes (the graduate competencies the overall curriculum is designed to achieve). Engage proactively with ethical responsibilities: transparency with learners, data privacy, algorithmic bias, overreliance on AI outputs, and ongoing monitoring after platform updates. Apply principles of fairness, accountability, and harm prevention as a standing framework.

a TPACK: technological pedagogical content knowledge.

This paper is a tutorial, not an evaluation study: its contribution is a practical, theoretically informed design workflow. Two locally developed initiatives, the Virtual Integrated Patient (VIP) and the Depression Avatars project, are drawn on throughout as illustrative implementation examples that motivated specific design decisions. They are presented as feasibility and acceptability experiences, not as evidence of educational effectiveness; the empirical claims should be read as illustrative rather than confirmatory.

## Section 1: Before You Build—Defining the Purpose, Learner, and Persona

### Defining Your Educational Purpose

The most consequential decision in chatbot design is made before any tool is opened: clearly defining what the chatbot is meant to achieve. Health educators should begin by articulating the specific learning outcomes the chatbot will address, the learner group it will serve, and the stage of learning at which it will be deployed (preclinical, clinical, or postgraduate).

Chatbots serve distinct educational functions (formative assessment, deliberate practice, reflective debriefing, or knowledge retrieval), and the design choices for each differ substantially. A chatbot intended to simulate a psychiatric history-taking encounter requires a very different configuration from one designed to quiz students on pharmacological mechanisms. Conflating these purposes in a single tool frequently results in a chatbot that serves neither well. The same principle applies across disciplines: a law educator designing a client interview simulation faces analogous choices about scope and function.

**Figure 1.**
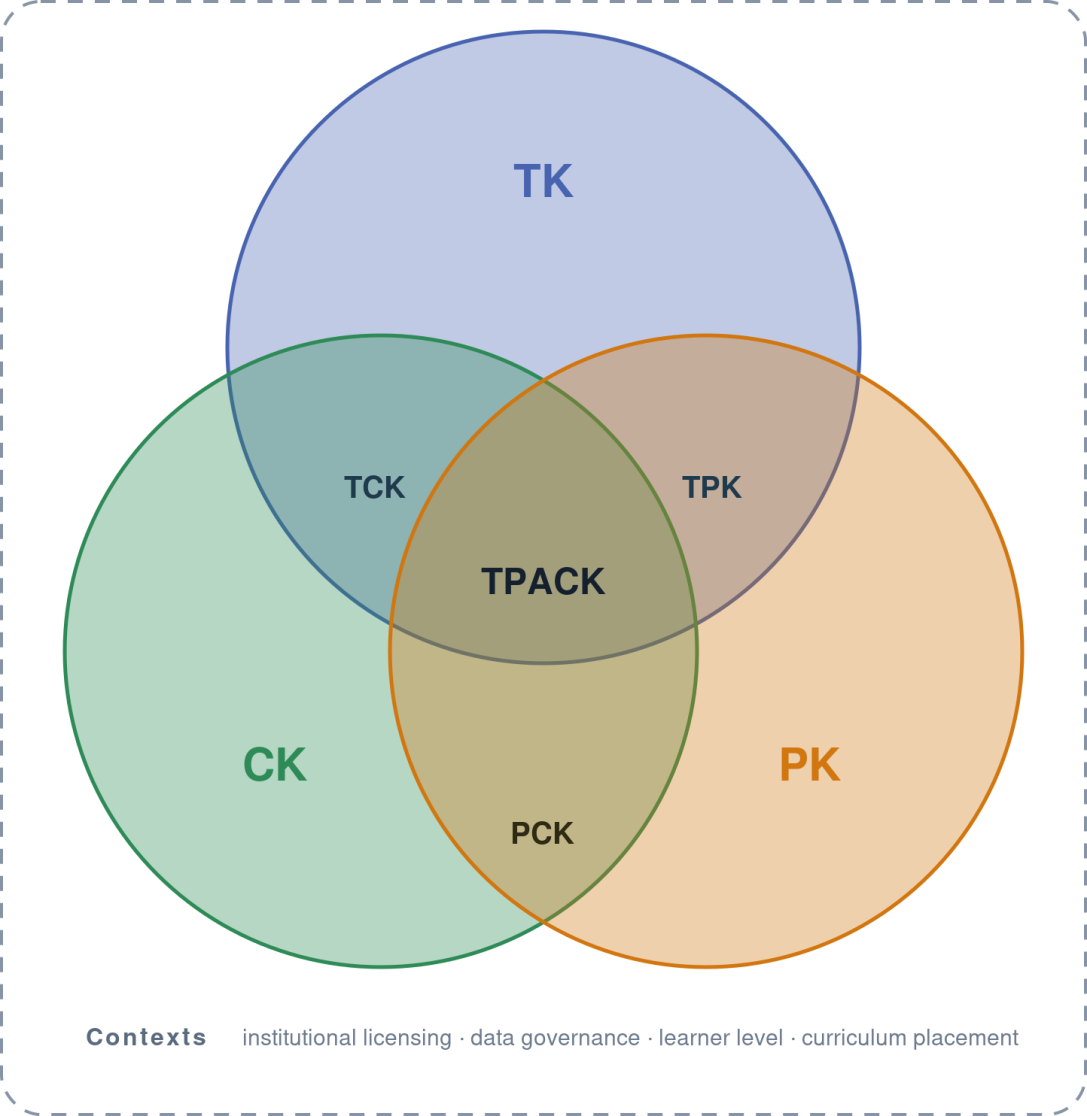
Application of the technological pedagogical content knowledge (TPACK) framework to no-code chatbot design.

The technological pedagogical content knowledge (TPACK) framework offers a useful heuristic at this stage [3]. Effective technology integration requires alignment among technological capability, pedagogical approach, and disciplinary content. Before building, the educator should be able to articulate clearly how the chatbot’s technological features support the chosen pedagogical strategy and reinforce the specific content domain (Figure 1).

While the TPACK framework explains what an educator must align, it does not by itself explain why the specific design choices in this workflow promote learning. Three established mechanisms provide that rationale and recur throughout the sections that follow. First, deliberate practice holds that competence develops through repeated, goal-directed effort on focused tasks with immediate feedback and progressive challenge [4]; the graduated disclosure and rehearsal properties of a well-designed chatbot (section 2) create exactly such focused, repeatable practice with consequences that are pedagogically informative rather than professionally harmful. Second, self-regulated learning describes how learners set goals, monitor their own performance, and adjust their approach [5]; structured, criterion-referenced feedback (section 2) supports this self-monitoring far more effectively than generic encouragement, which is why feedback is anchored to explicit objectives rather than offered impressionistically. Third, the simulation-based learning literature shows that technology-enhanced simulation improves knowledge and skill outcomes when it is integrated into a curriculum and used to supplement—not replace—authentic experience [6]; this is the basis for situating the chatbot within a planned instructional sequence (section 5) rather than deploying it in isolation. These mechanisms, together with the formative feedback evidence already noted, are what make the workflow pedagogically grounded rather than merely technically convenient.

### Defining the Chatbot’s Persona

Once the educational purpose is established, the chatbot requires a persona: a clearly specified identity that governs how it presents itself and communicates with learners. In health professions education, this most commonly takes the form of a simulated patient, a clinical tutor, a coaching companion, or an assessment examiner. In other disciplines, the persona might be a simulated client, a historical figure, a debating opponent, or a community stakeholder.

Persona specification should be detailed and explicit. For a virtual patient, this includes demographic information (age, gender, cultural background, occupation, and living situation), presenting concern, relevant medical and social history, personality characteristics, and communication style. In the Depression Avatars project, patient profiles were meticulously developed by psychiatric experts to ensure accurate representation of conditions, including adjustment disorder, dysthymia, major depressive disorder, panic disorder, and somatic symptom disorder, and scripts were localized to reflect Singapore’s cultural and health care context. This level of specificity produced clinically believable interactions and is directly traceable to the project’s positive learner evaluations.

The level of detail in persona design directly affects the authenticity and consistency of the chatbot’s responses. Vague personas produce generic outputs; richly specified ones produce interactions that challenge learners to think with contextual nuance regardless of the platform used.

## Section 2: Configuring the Chatbot—System Prompts, Disclosure, and Feedback

### Writing the System Prompt

In no-code LLM platforms, the system prompt is the primary mechanism through which an educator shapes chatbot behavior. It functions as standing instructions governing every learner interaction. This field is labeled differently across platforms (*Instructions* in ChatGPT’s custom GPT builder, *System prompt* in Claude Projects, and *Custom instructions* in Microsoft Copilot), but the function is identical: it tells the model who it is, what it knows, how it should behave, and what it must not do. Despite its centrality, it is frequently underestimated by first-time designers.

A robust system prompt for a clinical simulation chatbot should address at minimum (1) the role the chatbot will adopt; (2) the specific knowledge base it should draw upon; (3) interaction conventions, such as one question at a time and no unsolicited disclosures; (4) what the chatbot should not do; and (5) tone and language register.

An example instruction set for a simulated psychiatric patient might be as follows: “You will act as a patient based on the provided script. Your responses should be realistic, casual, and natural. Avoid volunteering information unless asked. Do not reveal pain or mood scale ratings unless explicitly requested. Use emotional expressions appropriate to the patient’s condition” [7]. Such specificity constrains the model toward clinically plausible, educationally productive responses and minimizes the risk of inappropriate information being offered spontaneously.

### Designing Graduated Information Disclosure

A common failure mode in novice chatbot design is configuring the system to behave as a cooperative, fully disclosing informant—a pattern that bears no resemblance to real clinical encounters and offers little educational challenge. In practice, patients disclose symptoms and history gradually, often require prompting, and do not volunteer information simply because a clinician is present.

Instructions should specify that the chatbot should reveal information incrementally, respond only to the question asked, and require the learner to exercise appropriate clinical communication techniques to elicit further details. Some patient personas may be appropriately designed as hesitant, anxious, or evasive, reflecting the genuine heterogeneity of patients encountered in practice. This principle applies equally beyond health care: a simulated legal client should not immediately disclose all relevant facts; an engineering stakeholder should not pre-empt a student’s needs analysis.

This design principle has direct pedagogical value: it rewards learners who ask open-ended, empathic, and systematically thorough questions and flags, through its silence, the omissions made by those who do not. The chatbot functions as a low-stakes rehearsal environment in which the consequences of incomplete questioning are pedagogically informative rather than professionally harmful [6].

### Building in Structured Feedback

Formative feedback is one of the strongest drivers of learning in health professions education [8], and a chatbot without a feedback function is a missed pedagogical opportunity. Feedback should be built into the instruction set from the outset rather than added as an afterthought.

Structured feedback frameworks such as the “what went well / what could be improved / try again” format provide a consistent and actionable response to learner performance. Critically, the feedback mechanism should be anchored to the specific learning objectives defined in section 1. If the objective is to assess the completeness of a psychiatric history, the chatbot’s feedback should explicitly address the components of a psychiatric history, not offer generic encouragement. This principle holds in any discipline: feedback should be criterion referenced, not impressionistic.

Effective feedback instructions might be as follows: “After each learner response, provide structured feedback identifying one specific strength, one area for improvement, and an invitation to attempt the response again. Do not reveal the model answer unless the learner explicitly requests it after two unsuccessful attempts. Use a supportive, non-judgmental tone and normalise mistakes as part of learning.” An end-of-session summary in which the chatbot identifies patterns across the full interaction further enhances the reflective value of the encounter. A recording demonstrating this structured feedback mechanism in practice is provided in Multimedia Appendix 2.

## Section 3: Refining the Experience—Learner Calibration, Voice, and Knowledge

### Calibrating Communication Style for Your Learner Group

Health professions learners span a wide range of experience levels, from preclinical students encountering patient communication for the first time to postgraduate trainees refining advanced clinical reasoning. A chatbot calibrated for a preclinical medical student using accessible language, offering frequent encouragement, and providing scaffolding may be inappropriate for a postgraduate trainee who requires diagnostic challenge. The same calibration imperative applies in any educational context.

Communication style calibration includes adjusting vocabulary, response length, degree of scaffolding, tolerance for incorrect responses before providing guidance, and the nature of prompts used to advance the interaction. For voice-enabled chatbots in particular, brevity is essential: voice interactions cannot replicate the visual scanning afforded by text, and lengthy responses overwhelm rather than inform.

Cultural calibration is equally important. Health communication norms vary significantly across cultures and health care systems. The Depression Avatars project deliberately localized patient scripts and speech patterns to reflect Singapore’s cultural and linguistic context. Educators should attend carefully to linguistic register and cultural assumptions embedded in patient personas before deploying to learners from different backgrounds.

### Using the Voice Function Deliberately

Many contemporary GenAI platforms offer voice interaction capability, enabling learners to speak to the chatbot and receive spoken responses. This substantially increases the ecological validity of clinical simulation, particularly for communication skill training, by requiring learners to engage in spoken, real-time dialogue rather than compose written text. In disciplines in which oral communication is central to professional practice, voice mode offers an authenticity that text cannot replicate.

Voice mode demands specific design constraints: responses must be short and conversational, lists and structured summaries are disorienting when spoken aloud, and conversation starters should be provided to reduce cognitive load at the start of an interaction. Educators must also verify institutional access: microphone permissions, browser compatibility, and network reliability vary significantly across learning environments and should be tested before voice mode is designated a required activity. An illustrative audio recording of a learner interacting with a voice-enabled chatbot is available as Multimedia Appendix 3.

### Supplementing With Curated Knowledge Documents

Most no-code GenAI platforms allow educators to upload supplementary documents (course materials, diagnostic criteria, clinical guidelines, rubrics, or patient case files) as a knowledge base from which the chatbot draws during interactions. This grounds the chatbot’s responses in curriculum-specific content rather than generic model outputs and enables alignment between chatbot feedback and formal assessment criteria.

Documents uploaded to the knowledge base should be concise, clearly structured, and directly relevant to the chatbot’s purpose. Uploading large, unstructured documents may degrade response quality. Educators should test chatbot outputs following each knowledge upload to verify that the intended material is appropriately influencing responses.

## Section 4: Adding Realism and Testing—Avatars, Multimodal Elements, and Pilot-Testing

### Adding Multimodal Elements to Increase Realism

Text-based chatbots are effective but limited in their ability to simulate the visual and emotional dimensions of professional encounters. Incorporating AI-generated avatar videos (photorealistic visual representations animated to match a generated script) can significantly enhance the perceived authenticity of simulated interactions. In the Depression Avatars project, patient scripts generated using GPT-4 were combined with avatar videos produced using the D-ID software, with facial expressions and speech synchronized to portray patients presenting with low mood and chest tightness. Student responses were positive, with most rating the avatar-based learning as more educationally useful than traditional formats [9]. A sample AI avatar patient video produced using these tools is available as Multimedia Appendix 4.

Educators can generate a visual patient persona from a descriptive text prompt alone. A prompt such as “A 50-year-old woman sitting in a clinic chair, wearing plain clothing, appearing slightly unkempt, hands folded in her lap, visibly anxious” can generate a photorealistic avatar that is then animated with the chatbot’s script. This approach combines the interactivity of the conversational chatbot with the visual richness of a video-based case study without specialist technical knowledge.

Educators should budget for multiple iterations before reaching a satisfactory output. The Depression Avatars project required several rounds of prompt refinement to achieve acceptable facial synchronization and emotional expressivity. Emotional expression and body language remain current limitations of avatar technology, but the investment in iterative refinement yields material improvement in the learning experience’s perceived authenticity.

### Pilot-Testing Rigorously Before Deployment

A chatbot that has not been tested before deployment risks exposing learners to factually inaccurate information, inappropriate responses, or unreliable interactions—all of which damage learner trust and educational outcomes. Predeployment testing is not optional regardless of the platform or discipline.

Pilot-testing should include structured review by a subject matter expert assessing content accuracy across a range of likely learner inputs. In the Depression Avatars project, psychiatric experts reviewed AI-generated patient profiles and scripts before student exposure to ensure clinical accuracy and cultural appropriateness. Expert review should be followed by testing with representative learners under realistic conditions, including voice mode if applicable.

Common failure modes to anticipate and test for include excessive unprompted disclosure of information, inaccurate disciplinary content, feedback that is too vague or poorly anchored to learning objectives, and persona inconsistency across extended interactions. Most failure modes can be addressed by adjusting the system prompt without platform-level changes. Simple additions such as “Do not volunteer information the learner has not asked for” or “Limit all responses to two to three sentences unless the learner explicitly asks for more” meaningfully improve output quality.

## Section 5: Embedding and Sustaining the Tool—Curriculum Integration and Ethics

### Situating the Chatbot Within a Broader Curriculum Framework

A persistent risk of educational technology adoption is deployment in isolation from the broader curriculum, reducing pedagogical impact and limiting transfer of learning. AI chatbots are most effective when situated within a planned instructional sequence that includes prelearning, supervised practical exposure, and structured reflection whether in a medical school, a law faculty, or a teacher education program.

Chatbots designed for deliberate practice function best when used after foundational content has been introduced through lectures or small-group teaching and before or alongside supervised real-world encounters. They are not a substitute for authentic professional experience; they function as a preparatory rehearsal environment that builds procedural fluency and reduces anxiety prior to higher-stakes settings, much as flight simulators prepare commercial pilots without replacing actual flight hours [4]. In the Depression Avatars project, avatar-based simulations were explicitly designed to supplement, not replace, psychiatric teaching and real patient contact.

Assessment integration strengthens the chatbot’s educational value further. Where chatbots support formative assessment, learner performance data (completeness of questioning, quality of reasoning, and responsiveness to feedback) may inform subsequent teaching priorities. Educators should map chatbot-based activities to program outcomes and establish a plan for monitoring learner progress over time.

### Engaging Proactively With Ethical Responsibilities

The deployment of AI chatbots in health professions education raises ethical responsibilities that must be addressed explicitly rather than deferred. These include data privacy, academic integrity, algorithmic bias, transparency with learners about AI involvement, and the risk of overreliance on AI-generated information. These concerns are not unique to health education; they are systemic issues wherever AI tools are deployed in learning environments.

Learners should be clearly informed that they are interacting with an AI tool, that its outputs may contain inaccuracies, and that professional decision-making must always be grounded in verified evidence and supervised practice. Safety messaging should be built into the system prompt directly: instructions such as “If uncertain, acknowledge uncertainty and direct the learner to consult a trusted clinical reference” reduce the risk of the chatbot being used as an authoritative source for consequential decisions.

Educators should attend to demographic and cultural biases embedded in the underlying LLM that may produce personas or scenarios that are unrepresentative or culturally insensitive. Ongoing monitoring of chatbot outputs is essential: AI systems evolve with platform updates, and a chatbot that performed appropriately at launch may require re-evaluation following changes to the underlying model. The principles of fairness, transparency, accountability, privacy, harm prevention, and trust building should serve as a standing framework for all AI-enhanced educational design [10].

## When Not to Use a Chatbot and Key Risks

No-code chatbots are not appropriate for every educational task, and being explicit about their limits is part of responsible design. They are poorly suited to high-stakes summative assessment, where the lack of reproducibility, auditability, and validated scoring makes them unfair as a basis for consequential decisions. They are a weak substitute for any competency whose core is authentic human interaction (breaking bad news, disclosing error, or responding to a distressed or angry patient) because current models do not reliably reproduce emotional variability, nonverbal cues, or sustained interpersonal tension; learners in the VIP evaluation made exactly this point (J. Sng, unpublished data, 2026). They should not be used in any workflow that requires entering patient-identifiable or otherwise confidential data into a consumer platform, and they are inadvisable where no faculty oversight, monitoring, or governance is available. Finally, chatbots carry particular risk for novice learners who lack the baseline knowledge to detect model error, where confidently stated inaccuracies may reinforce misconceptions rather than correct them.

The principal risks follow from these limits. Generative models can hallucinate plausible but incorrect clinical content; they can foster automation bias and overreliance that erodes independent reasoning; they raise data privacy exposure when learners paste real material; they can encode demographic or cultural bias into generated personas; and unequal access to paid tiers or institutional licenses can create equity gaps between learners. Several of these risks are not yet well quantified in health professions education, which is itself a reason to frame current tools as promising rather than proven and monitor them in use.

## A Practical Governance Checklist and the Conditions for Sustainable Use

General appeals to “privacy” and “transparency” are difficult to act on, so educators benefit from a concrete governance checklist. Before deployment, a minimum standard is to (1) define what data must never be entered into a public platform (real patient identifiers, uploaded clinical records, and any institution confidential material) and state this rule to learners in writing; (2) verify the platform’s data retention and training settings and prefer institutionally licensed tools with enterprise privacy terms where available; (3) assign a named owner responsible for monitoring outputs, retesting after model updates, and retiring the tool if quality degrades; (4) establish accountability for AI-generated error—the supervising educator, not the model, remains responsible for what learners are exposed to; and (5) train learners explicitly to interrogate AI outputs rather than accept them.

This last point reframes chatbot use as an opportunity rather than only a hazard. A central aim of deployment should be to move learners from uncritical acceptance toward verification and meta-cognitive engagement, treating the chatbot as a fallible interlocutor to be questioned, cross-checked against trusted references, and corrected [11]. Cultivating this critical AI literacy is arguably as valuable as the domain practice the chatbot provides, and it is a competency that learners will carry into clinical environments increasingly saturated with AI tools.

Sustainability, finally, depends on conditions beyond any individual educator’s enthusiasm. No-code tools lower the technical barrier, but they do not remove the need for educator training, institutional licensing and platform access, data governance approval, curriculum alignment, recognition of the workload involved in building and maintaining tools, and ongoing quality assurance; designing and supervising AI-mediated learning redistributes rather than simply reduces educators’ cognitive labor, with implications for professional judgment and reflective practice [12]. Where these enabling conditions are absent, even well-designed chatbots tend to remain isolated pilots rather than durable curriculum components. Educators are therefore advised to secure institutional support early, particularly given the high-stakes nature of professional preparation in health disciplines.

## Illustrative Implementation Examples

The workflow presented in this tutorial was developed through iterative experience building and evaluating 2 chatbot-based tools at the Yong Loo Lin School of Medicine, National University of Singapore.

The first, the VIP, is a free-text conversational platform in which an LLM is configured to simulate patients presenting with a range of clinical conditions for history-taking practice. Qualitative interviews with medical students (n=8) identified VIP as a flexible, self-directed tool valued for applying knowledge in simulated patient scenarios. Students noted limited emotional realism and the absence of visual cues as constraints on the depth of the experience and consistently emphasized that VIP functioned best as a supplement to, not a replacement for, traditional teaching and real patient contact (J. Sng, unpublished data, 2026).

The second initiative, the Depression Avatars project, sought to address these limitations by combining GPT-4–generated clinical scripts with photorealistic AI avatar videos produced using the D-ID software. Patient profiles spanning low mood (adjustment disorder, dysthymia, and major depressive disorder) and chest tightness (adjustment disorder, panic disorder, and somatic symptom disorder) presentations were developed by psychiatric experts, localized to Singapore’s clinical context, and presented to medical students as diagnostic exercises. Early feedback from medical educators and students suggested that the approach was engaging and acceptable, and a student pilot to gather quantitative and qualitative feedback was subsequently conducted; formal outcome data are not yet available [9]. These observations are reported as feasibility and acceptability experiences, not as evidence of learning gains.

The findings from both initiatives directly informed the 5-section workflow: VIP’s evaluation shaped the sections on graduated disclosure and curriculum supplementation; the Depression Avatars project informed the sections on persona specificity, cultural localization, avatar iteration, and expert-led pilot-testing. Rigorous evaluation using controlled study designs, validated outcome measures, and multi-institutional replication remains necessary before definitive conclusions about learning efficacy can be drawn.

## Conclusions

No-code AI chatbots represent a genuine opportunity to extend the reach and quality of experiential learning in health professions education, particularly where patient access is limited and contact time is constrained. The 5-section workflow presented in this paper, from defining the purpose and persona to configuring the interaction and refining for the learner to adding realism, testing rigorously, and sustaining the tool ethically, enables educators to move from intention to deployed chatbot with pedagogical rigor regardless of technical background.

While the primary audience is health professions educators, the workflow is transferable across higher education wherever interactive, scenario-based learning is valued. The tools listed in Table 1 will change; the design decisions summarized in Table 2 will not. As the AI landscape continues to evolve, sound instructional design remains the most durable foundation for effective chatbot development.

## Supplementary material

10.2196/103399 Multimedia Appendix 1 Step-by-step educator guide: building text-based chatbots, voice-enabled chatbots, and AI avatar patient videos without coding. Includes system prompt templates, platform selector table, troubleshooting guides, and expert review checklist.

10.2196/103399 Multimedia Appendix 2 Audio recording of a medical student practicing psychiatric history taking using a voice-enabled custom generative pretrained transformer chatbot with structured AI-generated feedback. The recording illustrates the “what went well / what to improve / try again” feedback mechanism described in section 2 of this guide. Student consent was obtained prior to recording. Identifying information has been removed.

10.2196/103399 Multimedia Appendix 3 Audio recording of a medical student practicing psychiatric history taking using a voice-enabled custom generative pretrained transformer chatbot. Student consent was obtained prior to recording. Identifying information has been removed.

10.2196/103399 Multimedia Appendix 4 Sample AI-generated avatar video produced using HeyGen depicting a simulated psychiatric patient presenting with low mood in a clinical consultation setting. The avatar was generated from a text-based patient profile and a clinician-reviewed script written using a large language model. The “AI” watermark (lower left) indicates AI-generated content and should be disclosed to learners prior to viewing.

## Abbreviations

GenAI: generative AI
TPACK: technological pedagogical content knowledge
VIP: Virtual Integrated Patient
