# Supplementary material for: Empowering Educators to Build AI Chatbots in Health Professions Education: Tutorial on a No-Code Design Workflow
Source: JMIR Med Educ. 2026 Aug 14;12:e103399. doi: 10.2196/103399 (PMC13476007; doi:10.2196/103399)
Supplement: Supplementary file 1 [file mededu-v12-e103399-s001.docx]

**Multimedia Appendix 1**

**Practical Guide to Building No-Code AI Chatbots for Health Professions Education**

*A Hands-On Resource to Accompany the Tutorial Article*

*Goh CXL, Wang E, Kong J, et al. Empowering Educators to Build AI Chatbots in Health Professions Education: Tutorial on a No-Code Design Workflow. JMIR Medical Education.*

**Multimedia Appendices: What is included with this tutorial**

Three supplementary files accompany this tutorial article. All are submitted as multimedia appendices to JMIR Medical Education.

| **Appendix** | **File** | **Description** | **Format** |
| --- | --- | --- | --- |
| **Multimedia Appendix 1** | This document | Step-by-step guide: text chatbot, voice chatbot, avatar generation | *Word* |
| **Multimedia Appendix 2** | Interaction with custom GPT.mp4 | Audio/video recording of a student practising with a voice-enabled chatbot | *MP4* |
| **Multimedia Appendix 3** | Feedback from custom GPT.mp4 | Audio/video recording of a student practising with a voice-enabled chatbot and receiving AI feedback | *MP4* |
| **Multimedia Appendix 4** | APMEC_Avatar.mp4 | Sample AI avatar video — simulated psychiatric patient (HeyGen, ~32 seconds) | *MP4 video* |

*This document (Multimedia Appendix 1) covers Parts 1–3 below. For the student voice practice recordings, see Multimedia Appendices 2 and 3; for the sample avatar video, see Multimedia Appendix 4.*

**What is in this guide**

Part 1 : Text-Based Chatbot (any LLM platform)

Part 2 : Voice-Enabled Chatbot (platforms with microphone support)

Part 3 : Avatar Generation (D-ID or HeyGen)

Each part includes: ready-to-copy prompts, step-by-step instructions, a troubleshooting table, and design tips.

No coding skills required. Estimated time: 20–30 minutes per part.

All prompts and instructions are platform-agnostic. A platform selector table on the next page

maps each step to your chosen tool (ChatGPT, Claude, Copilot, Gemini, DeepSeek, or Doubao).

# Platform Selector: Where to Find Each Feature

This guide uses generic terms throughout. Use this table to locate the equivalent feature on your chosen platform before starting.

| **Platform** | **Where to find it** | **System prompt field** | **Starters** | **Knowledge upload** | **Voice** | **Notes** |
| --- | --- | --- | --- | --- | --- | --- |
| **ChatGPT Custom GPT** | chatgpt.com → Explore GPTs → Create | Instructions | Conversation starters | Knowledge | Yes — microphone icon in chat | Paid plan for full GPT builder; free tier limited |
| **Claude Projects** | claude.ai → Projects → New Project | System prompt / Project instructions | — | Upload to Project files | No native voice | Excellent instruction-following; large context window |
| **Microsoft Copilot** | copilot.microsoft.com | Custom instructions (via system message) | Suggested prompts | Via SharePoint or uploaded files | Yes — microphone icon | Free via many institutional Microsoft licences |
| **Google Gemini Gems** | gemini.google.com → Gems | Instructions | Conversation starters | Upload files | Yes (mobile app) | Gems require paid plan; strong Google Workspace integration |
| **DeepSeek** | deepseek.com | System prompt in chat | Type manually | Paste into prompt | No | Free; open-source; verify data policies for your institution |
| **Doubao 豆包** | Mobile app (China) | Paste role instruction as first message | Type manually | Paste into conversation | Yes — microphone in app | Recommended for China-based educators and learners |

*Tip: If your institution restricts certain platforms, check with your IT department about approved alternatives. Microsoft Copilot is frequently available under existing institutional Microsoft licences.*

# Part 1: Building a Text-Based Chatbot

A text-based chatbot is the fastest entry point. In this part you will configure your chosen LLM platform to act as a simulated patient, clinical coach, or formative assessment partner. All instructions use generic terms mapped to the Platform Selector table above.

**Live example: MindMate**

MindMate is a Custom GPT simulated psychiatric patient developed at NUS Medicine for

student history-taking practice. You can try it directly using the QR code below,

or visit: chatgpt.com/g/g-YoAJz4Iq0-simulated-psychiatry-patient-for-students

(A ChatGPT account is required.)

*Figure 1. QR code linking to MindMate — a simulated psychiatric patient Custom GPT developed at the Yong Loo Lin School of Medicine, National University of Singapore. Scan to access the live chatbot. A ChatGPT account is required.*

**What you need before you start**

An account on your chosen LLM platform (see Platform Selector above)

Your chatbot's teaching purpose — e.g. 'TB history-taking practice for nursing students'

3–5 common learner mistakes or gaps in your topic area

One short scenario or case to test your chatbot after building it

## Step-by-Step: Configure Your Chatbot

**Step 1** Open the chatbot builder on your chosen platform

• Navigate to the chatbot or GPT creation area (see Platform Selector for exact location)

• Create a new chatbot, agent, Gem, or project as appropriate for your platform

• Give it a name that reflects its educational purpose — e.g. MindMates, TB Coach, History Partner

**Step 2** Write your system prompt — the most important step

The system prompt (variously called Instructions, System prompt, or Custom instructions depending on your platform) tells the chatbot who it is, how it should behave, and what it must not do. Copy and edit the template below:

**System Prompt Template — copy, paste, and edit the [brackets]**

```
ROLE
You are a [simulated patient / coaching chatbot / formative assessor] for
[learner group, e.g. first-year Master of Nursing students] learning [topic,
e.g. tuberculosis history-taking].

PERSONA (complete this section if building a simulated patient)
Your name is [patient name]. You are a [age]-year-old [gender], [occupation],
presenting with [presenting complaint]. You have [key history details].
Your communication style is [e.g. hesitant, mildly anxious, cooperative].
Do not volunteer information unless directly asked.

INTERACTION RULES
- Respond to one question at a time.
- Do not reveal information the learner has not asked for.
- Keep all responses to 2-3 sentences unless the learner asks for more.
- Do not provide scores or ratings unless explicitly asked.

FEEDBACK (provide after each learner response)
Use this format every time:
  What you did well: [one specific strength]
  What to improve: [one clear area for development]
  Try again: [invite the learner to reattempt or refine]
Do not give the model answer unless the learner explicitly asks after two attempts.

STYLE
Warm, concise, non-judgmental. Normalise mistakes as part of learning.
If unsure of a fact, say so and recommend checking a trusted clinical reference.

SAFETY
If any message raises concerns about patient safety or clinical error,
flag this clearly and recommend consulting a supervisor.
```

**Step 3** Add conversation starters

Most platforms allow you to add clickable opening prompts. These reduce learner hesitation and model the expected type of interaction. Add 3–4 from the suggestions below:

**Suggested conversation starters — edit [topic] to match your content**

"Start a history-taking practice case."

"Quiz me on the key symptoms of [topic]."

"Give me feedback on how I asked about this patient's social history."

"Help me revise the [topic] framework."

"Start a new case — I want to try a different patient."

**Step 4** Upload a knowledge document (optional)

• Upload a short, structured document: course rubric, case outline, checklist, or clinical criteria

• Keep it concise — large unstructured documents degrade response quality

• Test chatbot outputs after each upload to verify the document is influencing responses correctly

**Step 5** Save and test

• Save your chatbot with the appropriate sharing setting (private for testing; link-sharing for students)

• Open the chatbot and type one of your conversation starters

• Check: does it stay in persona? Does it disclose information gradually? Is feedback structured?

• Adjust the system prompt if any behaviour is incorrect — this is normal and expected

### End-of-Session Feedback Loop - add to system prompt if desired

**Optional: add this block to your system prompt for session-level feedback**

```
END-OF-SESSION SUMMARY
When the learner signals they have finished (e.g. 'I am done', 'end session'),
provide a brief summary:
  - One key strength demonstrated across the session.
  - One priority area to focus on in the next practice session.
  - An encouraging closing message.
```

## Troubleshooting: Text Chatbot

| **Problem** | **Fix — add to your instruction/system prompt field** |
| --- | --- |
| Chatbot talks too much | "Keep all responses to 2-3 sentences unless I explicitly ask for more." |
| Chatbot gives away answers too quickly | "Do not reveal the ideal answer. Ask guiding questions first." |
| Chatbot is too formal or robotic | "Use a warm, supportive tone. Normalise mistakes as part of learning." |
| Chatbot invents clinical facts | "If unsure of a fact, say so and recommend checking a trusted clinical reference." |
| Chatbot breaks character (simulated patient) | "Stay in character as [patient name] at all times." |
| Feedback is too vague | "Feedback must name one specific strength and one specific improvement." |

# Part 2: Adding Voice to Your Chatbot

Voice mode transforms a text chatbot into a spoken clinical conversation, significantly increasing ecological validity for communication skills practice. This part assumes you have completed Part 1 and have a configured chatbot ready to use.

**See Multimedia Appendix 3**

A recording of a student practising with a voice-enabled chatbot — including the

structured AI feedback response — is available as Multimedia Appendix 3.

The recording demonstrates the 'What went well / What to improve / Try again' feedback

mechanism described in this section. Student consent was obtained prior to recording.

**Before you start**

Complete Part 1 first — you need a configured chatbot

Check your platform supports voice (see Platform Selector: Voice column)

Use a desktop or laptop browser (recommended over mobile for first-time setup)

Enable microphone access when your browser or app prompts you

Test in a quiet space — background noise degrades voice recognition accuracy

## Step-by-Step: Activate and Use Voice Mode

**Step 1** Open your configured chatbot on your platform

**Step 2** Locate and activate the voice / microphone icon

• On most platforms this appears in the bottom-right of the chat interface

• Click Allow when your browser asks for microphone access

• On mobile apps (Gemini, Doubao), tap the microphone icon in the message bar

**Step 3** Test with these opening phrases

**Suggested opening phrases for voice testing**

"Start a practice case and guide me through history-taking — one question at a time."

"Please give me feedback after each answer I give."

"Begin. I am ready to take the patient history."

Expected behaviour: the chatbot should speak back in short turns, ask one question at a time, and coach rather than lecture. If it gives long responses, apply the troubleshooting fixes below.

## Voice-Specific System Prompt Addition

Add this block to your existing system prompt before using voice mode. It constrains the chatbot to spoken-conversation norms:

**Voice design rules — add these to your instruction/system prompt field**

Keep all responses under 2-3 sentences. Voice users cannot scan long text.

Ask only one question per turn. Wait for the learner to respond before continuing.

Avoid bullet points, numbered lists, and formatting marks — these are disorienting when spoken.

Use natural spoken language rather than formal written syntax.

Include at least one reflection prompt per session:

"What would you do differently next time?" or "What felt most challenging?"

**Voice Mode block — paste into your system prompt before using voice**

```
VOICE MODE RULES
This chatbot will be used in voice mode. Apply the following:
- Keep every response under 2-3 sentences.
- Ask exactly one question per turn. Do not ask multiple questions at once.
- Do not use bullet points, lists, or numbered steps.
- Use natural conversational language as if speaking directly to the learner.
- End each interaction with one reflection question:
  "What part of this felt most challenging?" or
  "What would you do differently next time?"
```

## Setting Up Voice on Doubao (豆包) — for China-Based Users

Paste the following as your first message in a new Doubao conversation to set the chatbot's role, then tap the microphone icon to begin voice interaction:

**豆包 Role-Setting Prompt (Chinese) — paste as first message, then activate microphone**

你是一名支持型教学助理，帮助【护理 / 医学 / 药学】学生学习【主题】。

你的目标是引导学生思考，而不是直接给答案。

请一次只问一个问题，并等待学生回答。

在每一次学生回答后，请按照以下结构给予反馈：

1. 做得好的地方（指出一个具体优点）

2. 可以改进的地方（指出一个关键点）

3. 请学生再尝试一次

不要立刻给出标准答案，除非学生明确要求。

语气应当鼓励、简短、非评判性。

*English meaning: You are a supportive teaching assistant helping [nursing / medical / pharmacy] students learn [topic]. Guide thinking rather than giving answers. Ask one question at a time and wait for the response. After each learner response, provide structured feedback: (1) what they did well; (2) one area to improve; (3) invite them to try again. Do not reveal the answer immediately. Use an encouraging, concise, non-judgmental tone.*

## Troubleshooting: Voice Chatbot

| **Problem** | **Fix — add to your instruction/system prompt field** |
| --- | --- |
| No microphone icon visible | Check Platform Selector — not all platforms support voice. On desktop, verify microphone permissions in browser settings. |
| Chatbot responds in text only | Look for a headphones or voice-conversation icon (not just the microphone for dictation). These are different modes. |
| Chatbot speaks too long | "In voice mode, keep every response under 2 sentences. Never use lists or bullet points." |
| Chatbot asks multiple questions at once | "Ask exactly one question per turn. Pause and wait for the learner to respond before continuing." |
| Feedback is not structured | Return to your system prompt and paste the full Feedback block from Part 1. Save and reopen the chatbot. |
| Voice session loses context | Voice sessions can time out on some platforms. Re-open and use a conversation starter to resume context. |

# Part 3: Generating an AI Avatar Patient

AI avatar tools combine a photorealistic image with a spoken script to create a video of a simulated patient. The avatar provides visual and emotional cues - facial expression, speech, body language - that text chatbots cannot replicate. This part covers a two-step workflow: (1) generate a patient script using any LLM, and (2) create the avatar video using D-ID or HeyGen. Both tools are interchangeable for this workflow; the original NUS Depression Avatars project used D-ID, while the sample video accompanying this guide (Multimedia Appendix 4) was produced with HeyGen.

**See Multimedia Appendix 4: Sample Avatar Video**

A 32-second sample avatar video — produced using HeyGen for the APMEC 2025 conference —

is available as Multimedia Appendix 4 (file: APMEC_Avatar.mp4).

The avatar depicts a simulated young adult female patient presenting in a clinical

consultation setting, wearing a patient gown, with a visibly anxious expression.

The video was generated from an LLM-produced script using the workflow described in this section.

The 'AI' watermark (lower left corner) is generated automatically by HeyGen to indicate

AI-generated content; this should be disclosed to learners prior to viewing.

*Figure 2. Still frame (at 10 seconds) from the sample avatar video used at APMEC 2025 and 2026 (Multimedia Appendix 4). The avatar depicts a simulated young adult female patient in a clinical consultation setting, generated using HeyGen from an LLM-produced patient script. The ‘AI’ watermark (lower left) is automatically applied by HeyGen to indicate AI-generated content and should be disclosed to learners before viewing. Full video available as Multimedia Appendix 4.*

**What you need before you start**

A patient profile: demographics, presenting complaint, key history, emotional state

Access to any LLM platform (for script generation : Part 1 platforms all work)

Access to D-ID (www.d-id.com) or HeyGen (app.heygen.com) : free trials available

Expert review: a subject matter expert must review the script before student use

Estimated time: 30-45 minutes for first avatar; faster with practice

## Step 1: Generate a Patient Script Using Your LLM

Use any LLM platform from Part 1 to generate a clinically accurate, culturally appropriate patient monologue from your patient profile. The script becomes the audio input for your avatar.

### Patient Profile Template

**Patient Profile Template — complete before generating the script**

Patient name: [e.g. Sarah Tan]

Age and gender: [e.g. 32-year-old woman]

Occupation: [e.g. primary school teacher]

Presenting symptom: [e.g. persistent low mood for 6 weeks]

Diagnosis category: [e.g. adjustment disorder with depressed mood]

Key history: [e.g. recent job change, supportive family, no past psychiatric history]

Emotional state: [e.g. mildly anxious, cooperative, tendency to minimise symptoms]

Cultural context: [e.g. Singapore; Chinese-Singaporean; prefers indirect communication]

Physical appearance: [e.g. neat, makes intermittent eye contact, speaks quietly]

### Script Generation Prompt — paste into any LLM platform

**Script Generation Prompt — paste into your LLM and edit [brackets]**

```
Generate a 2-minute spoken monologue for a simulated patient with the following profile.
The script will be used to produce an AI avatar video for health professions education.

Patient profile: [paste your completed profile above]

Requirements:
- Write in first person, as if the patient is speaking directly to a clinician.
- Reflect the patient's emotional state naturally — do not over-dramatise.
- Use culturally appropriate language and natural speech rhythms.
- Do not reveal the diagnosis explicitly — present symptoms and history only.
- Length: approximately 250-300 words (2 minutes of speech).
- End with the patient asking a natural closing question, e.g.:
  "Is this something serious, doctor?"

IMPORTANT: This script must be reviewed by a subject matter expert before use.
```

## Step 2: Generate the Avatar Video

Two platforms are recommended. Both support free trials. HeyGen was used to produce the sample video in Multimedia Appendix 4.

### Option A: HeyGen (recommended — used for APMEC sample)

| **Field** | **Instructions** |
| --- | --- |
| **Website** | app.heygen.com — free trial available; account registration required |
| **Step 1** | Log in and click Create Video → AI Avatar |
| **Step 2** | Choose an avatar from the library OR upload a photo to animate |
| **Step 3** | Paste your script into the Script field |
| **Step 4** | Select a voice matching your patient's age, gender, and cultural background |
| **Step 5** | Adjust speech speed to 0.9–1.0 (slightly slower aids comprehension) |
| **Step 6** | Click Generate and preview — review lip sync, expression, and speech clarity |
| **Step 7** | Download as MP4 and embed in your LMS or share via link |
| **Voice cloning (optional)** | Record 30+ seconds of your own voice; HeyGen can clone it for the avatar script |
| **Translation (optional)** | Upload an existing video and translate to another language with lip-sync |

### Option B: D-ID

D-ID allows you to generate an avatar from a text image prompt rather than selecting a pre-made avatar, giving more control over patient appearance.

| **Field** | **Instructions** |
| --- | --- |
| **Website** | www.d-id.com — free trial credits available |
| **Step 1** | Log in and click Create Video → Generate AI Presenter |
| **Step 2** | Generate an avatar image using the prompt template below |
| **Step 3** | Paste your script and select a matching voice |
| **Step 4** | Adjust emotion setting (Natural, Serious, or Surprised) to match patient presentation |
| **Step 5** | Generate and download as MP4 |

### Avatar Image Prompt Template (for D-ID or HeyGen photo-from-prompt)

**Avatar Image Prompt Template — edit to match your patient profile**

A [age]-year-old [gender] patient sitting on a chair in a clinic consultation room.

Wearing [clothing, e.g. plain casual clothes / patient gown].

Hands [e.g. folded on lap / resting on armrests].

Expression: [e.g. mildly anxious, making intermittent eye contact].

Photorealistic. Clinical background. Natural lighting. No text or watermarks.

Example (APMEC 2025 sample avatar):

A young adult East Asian woman sitting on a wooden chair in a clinic.

Wearing a light blue patient gown. Hands clasped in her lap.

Expression: visibly anxious, slight frown, looking directly at camera.

Photorealistic. Neutral clinical background. Natural lighting.

*Tip: Generate 3–4 image variations and select the most clinically plausible. Emotional expression and body language remain current limitations of avatar technology — expect to iterate.*

## Expert Review Checklist Before Student Deployment

**Verify all items before showing any AI avatar to students**

□ Clinical accuracy: a subject matter expert has reviewed the patient profile, script, and diagnosis category

□ Cultural accuracy: language, speech patterns, and context are appropriate for the target learner population

□ Emotional authenticity: the avatar's expression is plausible for the condition being portrayed

□ Consent and privacy: no real patient identifiers, photos, or recordings have been used

□ Disclosure: learners are clearly informed they are watching an AI-generated video, not a real patient

□ Task alignment: the diagnostic or history-taking task is clearly specified and mapped to learning objectives

□ Technical check: the video plays correctly in the LMS or sharing platform used for delivery

## Troubleshooting: Avatar Generation

| **Problem** | **Fix — add to your instruction/system prompt field** |
| --- | --- |
| Avatar looks uncanny or artificial | Add 'photorealistic' and 'natural lighting' to your image prompt. Generate 3–4 variants; select the best. |
| Lip sync is visibly off | Break long sentences into shorter ones in the script. Avoid complex consonant clusters at sentence ends. |
| Emotional expression is neutral or flat | Add specific emotion descriptors to the image prompt: 'slight tension around the eyes, downcast expression.' |
| Avatar appears wrong demographic | Be very explicit: include age, gender, ethnicity, and 2–3 specific physical descriptors in the image prompt. |
| Script sounds unnatural when spoken | Read the script aloud yourself before submitting. Adjust any phrase that feels awkward to say. |
| Students cannot access the video | Download as MP4 and upload to your institutional LMS. Do not share D-ID/HeyGen links directly to students. |

# Notes for Facilitators and Institutions

This guide was designed for use in faculty development workshops and as a companion resource to the JMIR Medical Education tutorial article. All prompts may be adapted freely for your institutional context.

**Before deploying any chatbot or avatar to students**

Pilot-test with a subject matter expert before student deployment

Inform learners clearly that they are interacting with AI-generated content

Monitor chatbot outputs after any platform update — model behaviour may change without notice

Store any student interaction data in compliance with your institution's data governance policies

Expect and plan for iteration: 2–3 rounds of system prompt refinement is normal and expected

Review your institution's policies on student consent for recordings before using Multimedia Appendix 3

**Contact and attribution**

For questions about this guide or the tutorial article:

Assoc. Prof. Judy Sng | phcsngj@nus.edu.sg

Department of Pharmacology, Yong Loo Lin School of Medicine

National University of Singapore

MindMate Custom GPT: chatgpt.com/g/g-YoAJz4Iq0-simulated-psychiatry-patient-for-students

Sample avatar video: Multimedia Appendix 4 (APMEC_Avatar.mp4)

Student voice practice recordings: Multimedia Appendices 2 and 3

# Source of Materials

The hands-on materials in this appendix, including the worked prompts, configuration templates, platform selector, voice-practice workflow, and avatar workflow, were developed and iteratively refined through a series of faculty-development workshops and invited lectures delivered by the author between 2025 and 2026. These presentations are listed below as the provenance of the materials reproduced here.

**1.** Sng J. Innovating psychiatric training through AI avatars and chatbot creation. Invited lecture; April 17, 2025; George Mason University, Virginia, USA.

**2.** Sng J. AI chatbots for faculty, students and deans: build your own without coding! Invited lecture, CenTILE AI Workshop in Health Professions Education; April 22, 2025; Georgetown University, Washington DC, USA.

**3.** Sng J. From concept to classroom: the journey of developing AI chatbots for medical education. Invited lecture; May 19, 2025; Vanderbilt University, Nashville, USA.

**4.** Sng J. No code, no fear: empowering educators to build AI chatbots for medical education. Invited lecture (online); July 18, 2025; University of California, Irvine, California, USA.

**5.** Sng J. AI in healthcare, neuroscience, and medical education: the evolution of the Virtual Integrated Patient (VIP). Invited lecture; November 1, 2025; Newcastle Medical University, Johor Bahru, Malaysia.

**6.** Sng J, Kong J, Dawe G. W2A7: build your own AI chatbot, without coding! Pre-conference workshop presented at: Asia Pacific Medical Education Conference (APMEC); 2026; Yiwu, China.
